# Structural Analysis of Botulinum Neurotoxins Type B and E by Cryo-EM

**DOI:** 10.3390/toxins14010014

**Published:** 2021-12-23

**Authors:** Sara Košenina, Markel Martínez-Carranza, Jonathan R. Davies, Geoffrey Masuyer, Pål Stenmark

**Affiliations:** 1 Department of Biochemistry and Biophysics, Stockholm University, 10691 Stockholm, Sweden; sara.kosenina@dbb.su.se (S.K.); markel.martinez-carranza@pasteur.fr (M.M.-C.); jonathanrd@gmail.com (J.R.D.); 2 Centre for Therapeutic Innovation, Department of Pharmacy and Pharmacology, University of Bath, Bath BA2 7AY, UK; 3 Department of Experimental Medical Science, Lund University, 22184 Lund, Sweden

**Keywords:** *Clostridium botulinum*, botulism, botulinum neurotoxin, BoNT/B, BoNT/E, cryo-EM

## Abstract

Botulinum neurotoxins (BoNTs) are the causative agents of a potentially lethal paralytic disease targeting cholinergic nerve terminals. Multiple BoNT serotypes exist, with types A, B and E being the main cause of human botulism. Their extreme toxicity has been exploited for cosmetic and therapeutic uses to treat a wide range of neuromuscular disorders. Although naturally occurring BoNT types share a common end effect, their activity varies significantly based on the neuronal cell-surface receptors and intracellular SNARE substrates they target. These properties are the result of structural variations that have traditionally been studied using biophysical methods such as X-ray crystallography. Here, we determined the first structures of botulinum neurotoxins using single-particle cryogenic electron microscopy. The maps obtained at 3.6 and 3.7 Å for BoNT/B and /E, respectively, highlight the subtle structural dynamism between domains, and of the binding domain in particular. This study demonstrates how the recent advances made in the field of single-particle electron microscopy can be applied to bacterial toxins of clinical relevance and the botulinum neurotoxin family in particular.

## 1. Introduction

The botulinum neurotoxins (BoNT) are potent bacterial toxins produced mainly by toxigenic *Clostridium botulinum* species [1]. Recently, several new BoNT and BoNT-like proteins have been discovered, considerably expanding this family of proteins beyond their traditional serotype classification [1,2]. The holotoxins are expressed as single-chain proteins that need to be proteolytically activated into a functional di-chain form, which consists of a ~50 kDa light chain (LC) linked by a single disulphide bond to the ~100 kDa heavy chain (HC) [3]. Although they all share a common modular architecture based on the three functional domains necessary for their activity, namely the binding (H_C_) and translocation domains (H_N_), which form HC and the catalytic (LC) domain, they present distinctive properties. In particular, variation in receptor recognition and substrate specificity dictate their species selectivity and pharmacological characteristics [4].

Serotypes A, B and E are the main BoNT associated with human botulism [5]. These neurotoxins specifically target presynaptic motoneurons via a dual-receptor binding mechanism, which involves membrane-anchored gangliosides [6,7,8] and a protein receptor [9]. BoNT/A, /D, /E and /F recognise the synaptic vesicle glycoprotein 2 (SV2), whereas BoNT/B, /G and /DC utilise one of the synaptotagmin isoforms [10,11,12,13,14,15,16,17]. Receptor-mediated endocytosis allow neuronal uptake via vesicular compartments where the acidic pH promotes translocation of the LC into the cytosol [18]. There, LC which is a zinc-protease, can degrade one of the soluble NSF attachment protein receptors (SNARE) responsible for exocytosis [19]. BoNT/A and /E cleave SNAP-25 (synaptosomal-associated protein of 25 kDa), BoNT/C targets both SNAP-25 and syntaxin, while BoNT/B, /D, /F, /G and /X degrade VAMP isoforms (vesicle-associated membrane protein), resulting in inhibition of neurotransmission and its associated flaccid paralysis [2,20].

Because of their extreme potency, BoNTs have also become the therapeutic molecules of choice for an increasing number of neuromuscular disorders, which include spasticity and dystonia, as well as other chronic conditions such as excessive sweating and migraines [21,22]. Two serotypes, BoNT/A (e.g., onabotulinumtoxinA, abobotulinumtoxinA) and /B (rimabotulinumtoxinB) are currently on the market and approved for therapeutic or cosmetic use. In addition, the variation in activity between natural BoNT serotypes translates into significantly different pharmacological profiles [23]; therefore, other BoNTs are being investigated as alternatives to currently available commercial products. For example, serotype E presents a faster onset of action and a shorter duration of action in humans [24], which may be beneficial for certain clinical applications that have been examined in several clinical trials [25,26].

**Figure 1:**
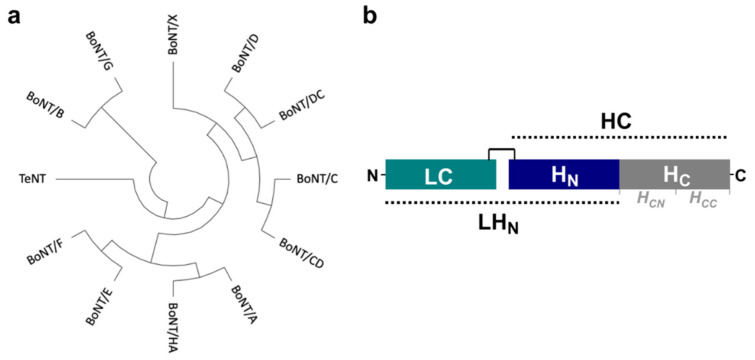
(**a**) Phylogenetic tree of clostridial neurotoxins prepared with MEGAX [33] from protein sequences aligned with CLUSTALO [34]. (**b**) Schematic representation of the botulinum neurotoxin domain structure. The toxins are expressed as single-chain proteins but are later proteolytically converted to their active di-chain form, where LC is linked by a single disulphide bridge to HC. The LH_N_ fragment has been defined previously as the combined LC + H_N_ domains [35].

BoNT/B and /E share approximately 40% sequence identity with BoNT/A, while homology can vary between 30 to 40% across other serotypes [3] (Figure 1). The first X-ray crystal structure of a BoNT holotoxin was that of type A [27], followed by BoNT/B [28] and /E [29]. Where the individual domains present identical folds, the overall architecture can vary between a linear configuration of the three functional domains, as seen in BoNT/A and /B, or the ‘closed’ arrangement seen in BoNT/E, where all three domains interact with each other in a conformation that is also associated with a faster translocation rate [30,31]. The structure of many of the single domains has been resolved by X-ray crystallography [3]. However, the full-length holotoxins remain challenging molecules to study with this method (Table 1), and so far, only low-resolution images, although informative, have been reported from single-particle microscopy [31]. The variation in interactions observed between domains and their effects on the toxins’ activity suggests that the toxins may present significant flexibility that is important to investigate. For example, structural studies on the closely related tetanus toxin demonstrated pH-mediated domain dynamics that are essential for its transport to the central nervous system and, thus, toxicity [32].

Here, we present the first cryo-EM structures of BoNT/B and /E by single-particle analysis. The fast development of this technique has made the study of BoNT in near atomic details more feasible. Our results show the subtle local dynamics within the toxins structure, which help us understand their complex mechanism of action. This study demonstrates that cryo-EM is a method of choice to pursue the structural characterisation of full-length botulinum neurotoxins.

## 2. Results and Discussion

### 2.1. Single Particle Analysis by Cryo-Electron Microscopy

#### 2.1.1. Data Collection and Analysis

**Figure 2:**
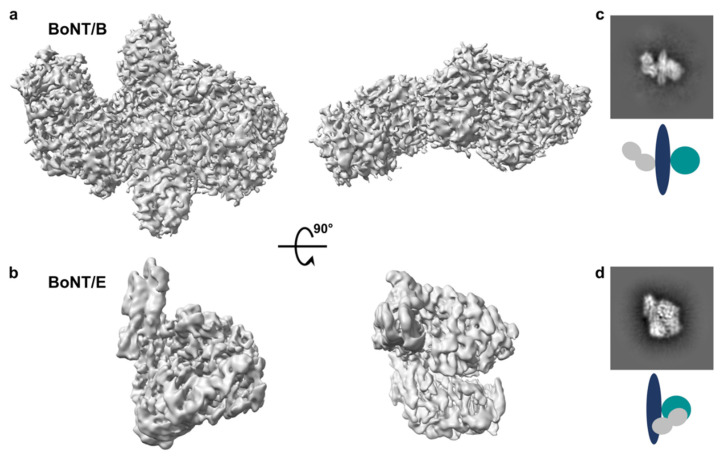
(**a**,**b**) Cryo-EM maps of BoNT/B and BoNT/E at an estimated resolution of 3.6 and 3.7 Å, respectively. The maps, as well as comparative 2D class averages of the toxins in (**c**,**d**), reveal clear secondary elements and domain arrangements illustrated by a schematic diagram (LC in green, H_N_ in blue, H_C_ in grey).

Botulinum neurotoxins consisting of catalytically inactive variants with a poly-histidine tag were recombinantly produced in *E. coli* and purified to homogeneity using traditional chromatography methods. For each sample, multiple conditions were screened with particle distribution appearing constantly most suitable on glow-discharged holey carbon copper grids. Complete movies were collected on a Titan Krios microscope, resulting in EM maps at 3.6 and 3.7Å average resolution for BoNT/B and BoNT/E, respectively, although local resolution varied significantly (Supplementary Figures S1 and S2). The map for BoNT/B appeared overall more precise than that of BoNT/E (Figure 2), even though image processing showed a slight preferential orientation for BoNT/B, which may partially explain the variation in local resolution. For both toxins, particle sizes were consistent with monomers, and secondary structure could clearly be distinguished in the 2D average classification, so that typical features of the individual domains were identifiable. Noticeably, the map quality around the binding domain was generally weaker compared to the rest of the toxin.

#### 2.1.2. Fitting of Protein Coordinates

**Figure 3:**
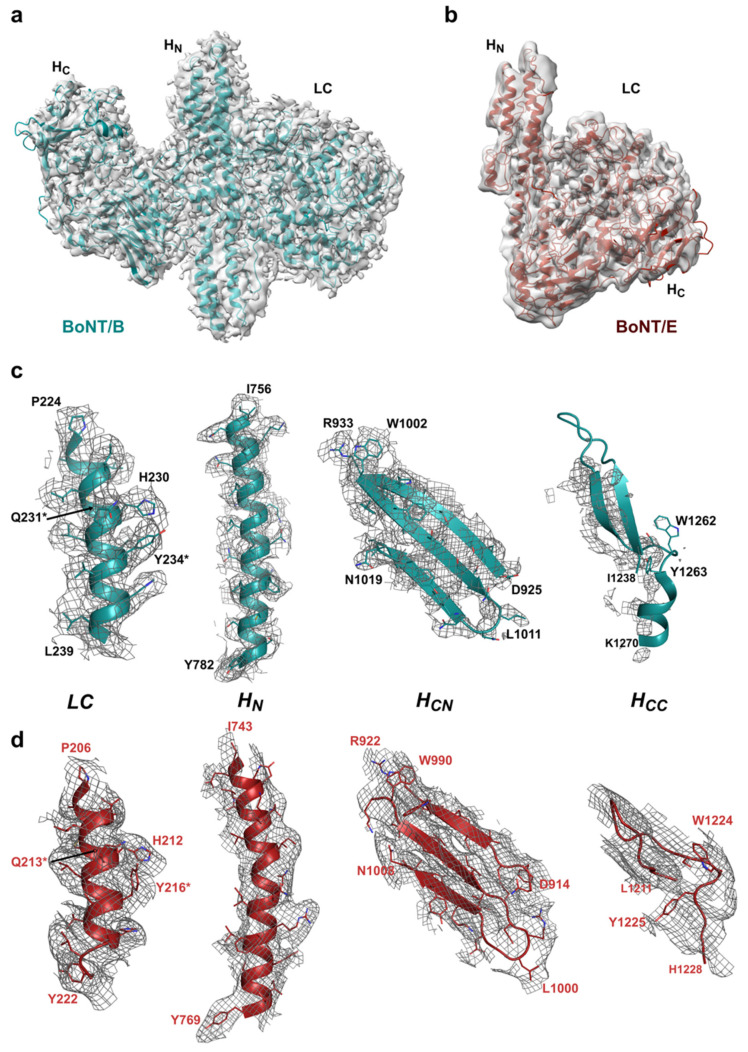
(**a**,**b**) 3D reconstruction of BoNT/B (teal) and BoNT/E (red), respectively. (**c**,**d**) Close-up map of secondary features from each domain, including the mutated catalytic site (mutations marked by *, E231Q/H234Y and E213Q/H216Y for BoNT/B and BoNT/E, respectively); α-helix of H_N_, β-sheet of H_CN_, and the ganglioside-binding site of H_CC_.

Cryo-EM maps of BoNT/B and /E confirmed the previously determined crystallographic structures of these holotoxins, which first revealed their different architecture [28,29]. Electron microscopy is often described as offering close to native structure. Here, BoNT/B also presents a linear arrangement of its three domains, whereas the BoNT/E map shows it retained its more compact domain organisation (Figure 3), thus alleviating reservation that this singular domain organisation among BoNTs may have been experimental artefacts [29,31]. Although the overall folds were similar, domains had to be fitted individually as rigid bodies to account for the observed local conformation variability. The domains were first docked in the cryo-EM maps with ChimeraX [36] before further real-space refinement on the whole toxins using the Phenix package [37].

The map is particularly well defined for the light chain (Supplementary Figures S1 and S2) and shows that all secondary structure elements are conserved. Despite the disorder observed for some of the larger flexible loops, the resolution obtained was sufficient to confidently assign side chains, particularly around the active site (Figure 3). Our structures are of inactive variants of the toxins presenting dual mutations at the catalytic site (BoNT/B1 E231Q/H234Y and BoNT/E1 E213Q/H216Y) [38]. LC is a metalloprotease, which normally contains a catalytic Zn^2+^ ion coordinated by the conserved HExxH motif [39]. A similar set of mutations (i.e., HQxxY) in BoNT/A was previously shown to prevent binding of the catalytic Zn^2+^ ion [40], which is also not visible in either of the two maps presented here. It should, however, be noted that whilst X-ray crystallography can precisely locate and assign protein-bound metal ions using X-ray fluorescence and anomalous diffraction [41], images collected with a standard transmission electron microscope, which are based on bright-field phase contrast, do not have sufficient sensitivity to identify isolated metal atoms [42].

The translocation domain (H_N_), which represents the central feature consisting of two long (approximately 100 Å), coiled-coil helices flanked by shorter α-helices is relatively well-defined. The resolution observed in the core region of H_N_ provided enough details to discern side chains, although the map definition became less precise in the polar parts of the domain (Figure 3). In addition, the so called ‘belt region’, which is an integral part of H_N_ at the primary sequence level, wraps around LC and closely interlocks the two domains together to stabilise their interaction, possibly acting as a protective chaperone for the holotoxins [43]. In the cryo-EM map of BoNT/B, the belt is well-ordered except for a small surface-exposed α-helix (residues 487–495; Supplementary Figure S3), whereas in BoNT/E, a section could not be modelled due to the lack of map for residues 499–515, which forms a random coil in the crystal structure.

For both toxins, the position of the binding domain (H_C_) was clear; however, the maps could not be interpreted in as much detail (Figure 3) and should, thus, be analysed with caution. This may be due to several factors. For BoNT/B, the lack of density in some areas, particularly the H_CC_ subdomain, suggests that the preferred particle orientation observed in the angular distribution map (Supplementary Figure S1) is the main reason affecting map quality and resolution, although local dynamism of the domain cannot be excluded. Such effect might be compensated by collection of larger datasets with altered parameters or grid preparation. However this kind of optimisation depends on a balance between the experimental goal and resource availability. Since H_C_/B has been well defined by X-ray crystallography, it was fully included in the deposited coordinates associated with the map.

In the case of BoNT/E, the map around H_C_ is also generally of lower resolution (Supplementary Figure S2), resulting in a blurrier picture. The main protein chain could be positioned, but it was difficult to carry out a stringent real-space refinement. Again, availability of the crystal structure for this domain allowed us to include it fully in our cryo-EM model. Remarkably, serotype E is the only BoNT in which the domain has so far been seen in a closed configuration [3]. Furthermore, the crystal structure of the BoNT/E–NTNH (progenitor M) complex [44] had shown that H_C_/E could take on a different position with a 60° rotation relative to the other two holotoxin domains (LC + H_N_) in a conformation stabilised by its interaction with NTNH and mediated by a flexible helical linker (BoNT/E residues 830–845) [29]. Observations from the cryo-EM map suggest H_C_/E is inherently dynamic thanks to the flexible linker with H_N_, which would affect the local resolution even though it does not imply a significant conformational change like the one observed in the progenitor complex.

### 2.2. Comparison with X-ray Crystal Structures 

**Figure 4:**
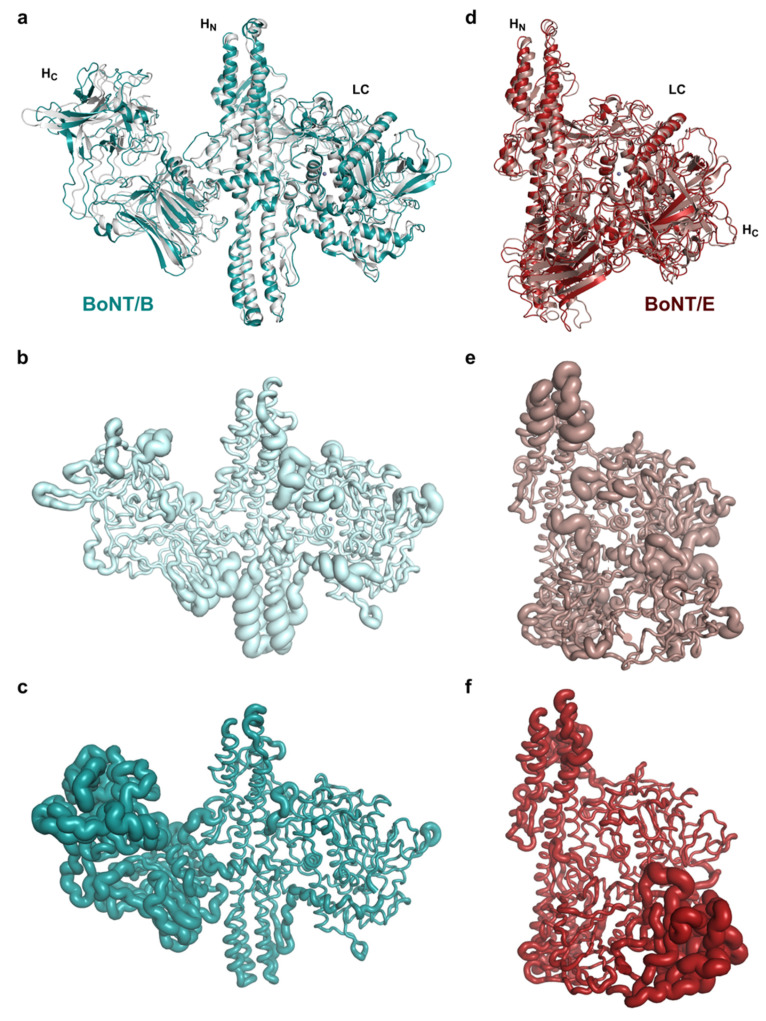
(**a**) Superposition of the cryo-EM (teal) and crystal (PDB 1EPW, light grey) coordinates of BoNT/B. (**b**,**c**) B factor-scaled ribbon representations of the crystal and cryo-EM structures of BoNT/B, respectively. (**d**) Superposition of the cryo-EM (red) and crystal (PDB 3FFZ, light pink) coordinates of BoNT/E. (**e**,**f**) B factor-scaled ribbon representations of the crystal and cryo-EM structures of BoNT/E, respectively.

Although the crystal structures of BoNT/B and BoNT/E were used to produce the cryo-EM models as described above, some differences were observed when comparing the output from both methods (Figure 4), with direct superposition showing root mean square deviations [45] of 1.9 Å (over 1218 paired residues) and 2.0 Å (over 1197 paired residues) for types B and E, respectively.

The main structural variations seem to come from overall movement of the domains with respect to each other. For example, overall superposition with BoNT/B results in a nearly perfect overlap of the translocation domains, whilst the core secondary structure of LC appears shifted by approximately 1.5 Å, with some of the larger loops deviating more significantly. On the other side of H_N_, the binding domain presents a more pronounced shift of up to 2 Å around the H_CN_ lectin-like subdomain, which seems to be further accentuated at the H_CC_ β-trefoil fold, although the cryo-EM map was weaker in that area (Figure 3) and should not be over-interpreted. Interestingly, BoNT/B presents a small helical linker between H_N_ and H_C_, which is similar to the one observed in BoNT/E [29]. Although no conformational changes have been reported for BoNT/B so far from several crystal structures solved at pH ranging from 4.0 to 7.0 [46], presence of this linker and the slight mobility observed within the cryo-EM map suggest that the binding domain could be subject to conformational changes when part of the progenitor complex with its NTNH partner, similarly to what has been observed with serotypes E [44] and A [47].

For BoNT/E, similar observations could be made with regards to the overall domain superposition, with slight shifts in domain position observed between the cryo-EM and crystal structures. Of note, part of the belt region, located in a wide crevice between two loops of LC, seems to be particularly mobile (Supplementary Figure S3). In addition, a small segment (residues 461–465), which is, itself, missing from the crystal structure, was visible in the cryo-EM map, highlighting the potential complementarity of the two biophysical methods. 

A different approach to compare the two techniques is to analyse the resulting structures looking at their atomic B factor. The definition of the B factor term is very different between X-ray crystallography and cryo-EM; however, in each case, it provides a means to measure the disorder and, to some extent, the local dynamism in the structure, so they can be compared qualitatively [48]. Figure 4 provides an illustration of the B factors analysis for BoNT/B and BoNT/E structures emanating from the two methods, with higher B factor representing a higher state of disorder. One caveat to that analysis for crystallography is that stability of the molecules is also significantly influenced by the crystal packing and quality. Nonetheless, solvent-accessible loop regions typically show higher B factors, and this can be observed in LC, the belt region, and H_C_ domains of the crystal and cryo-EM structures for both serotypes. Remarkably, the polar extremities of the H_N_ helices also present higher B factor in the crystal structures despite appearing clearly in the cryo-EM maps. One of the main differences resides in the binding domain, which is very well defined in the crystallographic structures but lacks resolution in the cryo-EM maps presented here. This represents a clear limitation to the method, and improvements need to be made so that atomic resolution can be reached to look at specific structural questions such as toxin–receptor or toxin–substrate interactions, which have eluded crystallography so far.

Cryo-electron microscopy has become a method of choice to determine the structure of macromolecules at near atomic resolution in close to native conditions, particularly for large and dynamic molecules for which crystallisation remains a challenge. However, cryo-EM is still limited by the size of smaller molecules and resolution obtained due to particle heterogeneity, which results in maps with high variability. Although these hurdles have hindered the obtention of high-resolution images for botulinum neurotoxins, the technique can provide a unique insight to study their molecular dynamism and catch multiple conformations that may be essential to understand the mechanism of action of BoNTs. This, for example, allowed the discovery of significant pH-mediated conformational changes in the related tetanus toxin [32]. The recent release of a database of AlphaFold-predicted models [49] for proteins of interest included several botulinum neurotoxins. Remarkably, whilst BoNT/E retains the domain organisation described in our study, BoNT/B appears incorrectly predicted with a shift in the binding domain position not observed in any experimental data (Supplementary Figure S4). Structural predictions are valuable but should be used cautiously and concurrently with experimental methods to determine accurate protein structures.

The single particle cryo-EM maps of BoNT/B and /E presented here provide a complementary perspective on the molecular structure of these toxins. In particular, it highlights the local mobility of each domain, as well as confirms unambiguously the structural information that had been obtained by X-ray crystallography. With the recent discovery of multiple BoNT and BoNT-like proteins, cryo-EM provides a useful biophysical technique to gain essential information on the mechanism of action of this powerful toxin family. Our study provides a methodological framework to analyse the atomic structure of botulinum neurotoxins and may help the design of novel antitoxin strategies or the development of new BoNT of clinical relevance. 

## 3. Materials and Methods 

### 3.1. Constructs

DNA encoding nontoxic, enzymatically inactive variants of BoNT/B1 (E231Q/H234Y) [38] (strain: Okra, residues 1 to 1291, UniProtKB: B1INP5) was kindly provided by Ipsen (Abingdon, UK) into a modified pET32a(+) vector in fusion with a C-terminal poly-His (6x) tag.

BoNT/E1 inactive variant (E213Q/H216Y) (strain: Beluga, residues 1 to 1252, UniProtKB: A8Y875) was synthesised by Genscript (Leiden, Netherlands) and cloned into a modified pET28a(+) vector designed to express the protein in fusion with a N-terminal 10xHis-tag, and TEV protease cleavage site.

### 3.2. Protein Expression and Purification

Both proteins were expressed in TB media inoculated using BL21(DE3) cells (New England Biolabs, Ipswich, MA, USA) transformed with the respective vector. Cultures were grown in a LEX bioreactor (Epiphyte Three Inc., Toronto, ON, Canada) at 37 °C. When the OD600 reached 0.8, the temperature was reduced to 18 °C, and protein expression was induced through the addition of 1 mM IPTG. Cells were grown for a further 18 h before harvesting by centrifugation. All chemical reagents were purchased from Sigma-Aldrich (Stockholm, Sweden).

For BoNT/B, cells were resuspended in 20 mM TRIS pH 7.5, 200 mM NaCl, 25 mM Imidazole and lysed using an Emulsiflex-C3 (Avestin Europe, Mannheim, Germany) at 20 kPsi. The lysate was clarified by centrifugation at 50,000× *g* for 45 min before loading onto a pre-equilibrated 5 mL HisTrap FF column (Cytiva, Uppsala, Sweden). Purified protein was eluted using a step elution with 20 mM TRIS pH 7.5, 200 mM NaCl, 250 mM Imidazole. Fractions containing BoNT/B1 were pooled and further purified using a Superdex200 26/600 column (Cytiva, Uppsala, Sweden) pre-equilibrated using 25 mM TRIS pH 7.5, 200 mM NaCl, 5% Glycerol. Protein was concentrated to 15 mg/mL with Vivaspin filters (10 kDa cut off, Sartorius, Goettingen, Germany). 

For BoNT/E, cells were resuspended in 50 mM Hepes pH 7.2, 200 mM NaCl, 25 mM Imidazole and lysed by sonication. The lysate was clarified by centrifugation at 42,000× *g* for 1 h before loading onto a pre-equilibrated 5 mL HisTrap FF column (Cytiva, Uppsala, Sweden). Purified protein was eluted using a step elution with 50 mM Hepes pH 7.2, 200 mM NaCl, 250 mM Imidazole, and 5% glycerol. Fractions containing BoNT/E were pooled and further purified using a Superdex200 16/600 column (Cytiva, Sweden) and pre-equilibrated using 50 mM Hepes pH 7.2, 200 mM NaCl, 5% Glycerol. Protein was concentrated to 1.4 mg/mL with Vivaspin filters (100 kDa cut off, Sartorius, Goettingen, Germany). 

Final concentration was measured by absorbance at 280 nm (NanoDrop Spectrophotometer, Thermo Fisher Scientific, Göteborg, Sweden), and proteins were flash frozen in liquid nitrogen for storage at −80 °C until further use.

### 3.3. Single Particle Cryo-Electron Microscopy

#### 3.3.1. Cryo-EM Grid Preparation

BoNT/B was prepared with an additional size exclusion chromatography step using a Superdex200 10/300 column (Cytiva, Uppsala, Sweden) pre-equilibrated in 20 mM HEPES pH 7.5, 200 mM NaCl, 0.5 mM TCEP. Sample at 0.05 mg/mL in 20 mM HEPES pH 7.5, 50 mM NaCl was pipetted onto glow-discharged holey carbon cryo-EM grids (Quantifoil Cu R0.6/1) and frozen in liquid ethane using a Vitrobot (Thermo Fisher Scientific, Göteborg, Sweden).

BoNT/E was prepared with an additional size exclusion chromatography step using a Superdex200 10/300 column (Cytiva, Uppsala, Sweden) pre-equilibrated in 20 mM HEPES pH 7.2, 200 mM NaCl, 1 mM TCEP. Sample at 0.1 mg/mL in 20 mM HEPES pH 7.5, 50 mM NaCl was pipetted onto glow-discharged holey carbon cryo-EM grids (Cu R1.2/1.3, Quantifoil, Großlöbichau, Germany) and frozen in liquid ethane using a Vitrobot (Thermo Fisher Scientific, Göteborg, Sweden).

#### 3.3.2. Cryo-EM Imaging

Cryo-EM experiments were conducted at the Swedish Cryo-EM National Facility, Umeå and Stockholm nodes (Sweden). Movies were collected in a Titan Krios (Thermo Fisher Scientific, Göteborg, Sweden) microscope operating at 300 kV in electron-counting mode and at a nominal magnification of 130,000× (1.09 Å/px) and 165,000× (0.86 Å/px) with an electron flux of 57.3 e/Å^2^ and 55 e/Å^2^ over 40 frames for BoNT/B and BoNT/E, respectively. The data were recorded using a Gatan K2 Summit (AMETEK, Leicester, UK) direct detector coupled with a Bioquantum energy filter, with a 20 eV slit.

#### 3.3.3. Cryo-EM Data Processing

For BoNT/B the frames were aligned, averaged, and dose-weighted in cryoSPARC [50]. CTF estimation and downstream processing were also carried out in cryoSPARC. A total of 2252 movies were recorded, from which a total of 2,432,309 particles were automatically picked, and after 2D classification, 676,677 particles were selected and used for 3D refinement, yielding the final map at 3.6 Å resolution, calculated based on the gold standard FSC of 0.143. The density map was sharpened by applying a negative B factor.

For BoNT/E the frames were aligned, averaged, and dose-weighted in cryoSPARC [50]. CTF estimation and downstream processing were also carried out in cryoSPARC. A total of 18,282 movies were recorded, from which a total of 1,096,741 particles were automatically picked, and after 2D classification, 284,390 particles were selected and used for 3D refinement, yielding the final map at 3.7 Å resolution, calculated based on the gold standard FSC of 0.143. The density map was sharpened by applying a negative B factor.

#### 3.3.4. Model Building and Validation

Protein validation was performed with MolProbity [51]. Data statistics are summarized in Table 2. The atomic coordinates and cryo-EM maps were deposited in the Protein Data Bank (PDB) with ID 7QFQ and 7QFP and the Electron Microscopy Data Base (EMDB) with ID 13947 and 13946 for BoNT/B and BoNT/E, respectively. Protein structure figures were rendered with PyMOL (Schrödinger, LLC, New York, NY, USA) or ChimeraX [36].

## Figures and Tables

**Table 1:** Overview of structural information available on clostridial neurotoxins.

	LC	LH_N_	H_C_	Full-Length	BoNT-NTNH
**BoNT/A**	1XTF	2W2D	2VU9	3BTA	3V0B
**BoNT/B**	2ETF	2XHL	1Z0H	1EPW	-
**BoNT/C**	1QN0	-	3N7K	-	-
**BoNT/CD**	*(=C)*	-	3PME	-	-
**BoNT/D**	2FPQ	5BQN	3OGG,3N7J,3OBR	-	-
**BoNT/DC**	*(=D)*	*(=D)*	3AZW	-	-
**BoNT/E**	1T3A	7K7Y	7OVW	3FFZ	4ZKT
**BoNT/F**	2A8A	-	3FUQ	-	-
**BoNT/G**	1ZB7	-	3MPP,2VXR	-	-
**BoNT/HA**	6BVD	-	5V38	-	-
**BoNT/X**	6F47	-	-	-	-
**TeNT**	1YVG	-	1AF9	5N0B	N/A

Nonexhaustive list of Protein Data Bank records, first-released entries included in this table. Brackets indicate when a domain is similar to another serotype in mosaic toxins.

**Table 2:** Cryo-EM Data Collection and Refinement.

	BoNT/B	BoNT/E
**Data collection and processing**		
Nominal magnification	130,000	165,000
Voltage (kV)	300	300
Electron exposure (e/Å^2^)	57.3	55
Defocus range (µm)	−1.9–−3.5	−0.5–−3
Pixel size (Å)	1.09	0.86
Number of images	2252	18,282
Symmetry imposed	C1	C1
Particles picked	2,420,309	1,096,741
Particles refined	286,802	284,390
Map resolution (Å)	3.6	3.7
FSC threshold	0.143	0.143
Map sharpening B factor	−151.9	−100
**Refinement**		
Initial model used (PDB code)	1EPW	3FFZ
Model composition		
Nonhydrogen atoms	10,660	9996
Protein residues	1291	1233
Ligand	0	0
B factors (Å^2^)	160	54
R.m.s.d. Bond lengths (Å)	0.002	0.007
R.m.s.d. Bond angles (°)	0.552	1.118
**Validation**		
MolProbity score	1.82	2.82
Clash score	8.21	18.19
Poor rotamers (%)	0	6.2
Ramachandran statistics:		
Favoured (%)	94.5	86.6
Outliers (%)	0	0.5
**PDB/EMDB ID**	7QFQ/13947	7QFP/13946

## Data Availability

The atomic coordinates and cryo-EM maps have been deposited in the Protein Data Bank (PDB, https://www.rcsb.org/, accessed on 5 December 2021) and the Electron Microscopy Data Base (EMDB, https://www.ebi.ac.uk/emdb/, accessed on 5 December 2021).

## Supplementary Materials

The following are available online at https://www.mdpi.com/article/10.3390/toxins14010014/s1: Figure S1: Image processing and evaluation of BoNT/B dataset; Figure S2: Image processing and evaluation of BoNT/E dataset; Figure S3: Belt region cryo-EM map. Figure S4: AlphaFold model predictions.
