# Supplementary material for: Structural Analysis of Botulinum Neurotoxins Type B and E by Cryo-EM
Source: Toxins (Basel). 2021 Dec 23;14(1):14. doi: 10.3390/toxins14010014 (PMC8781748; doi:10.3390/toxins14010014)
Supplement: Supplementary file 1 [file toxins-14-00014-s001.zip › toxins-1520391-supplementary.pdf]

Supplementary Materials: Structural Analysis of Botulinum Neurotoxins Type B and E by cryo-EM

Sara Košenina, Markel Martínez-Carranza, Jonathan R. Davies, Geoffrey Masuyer and Pål Stenmark

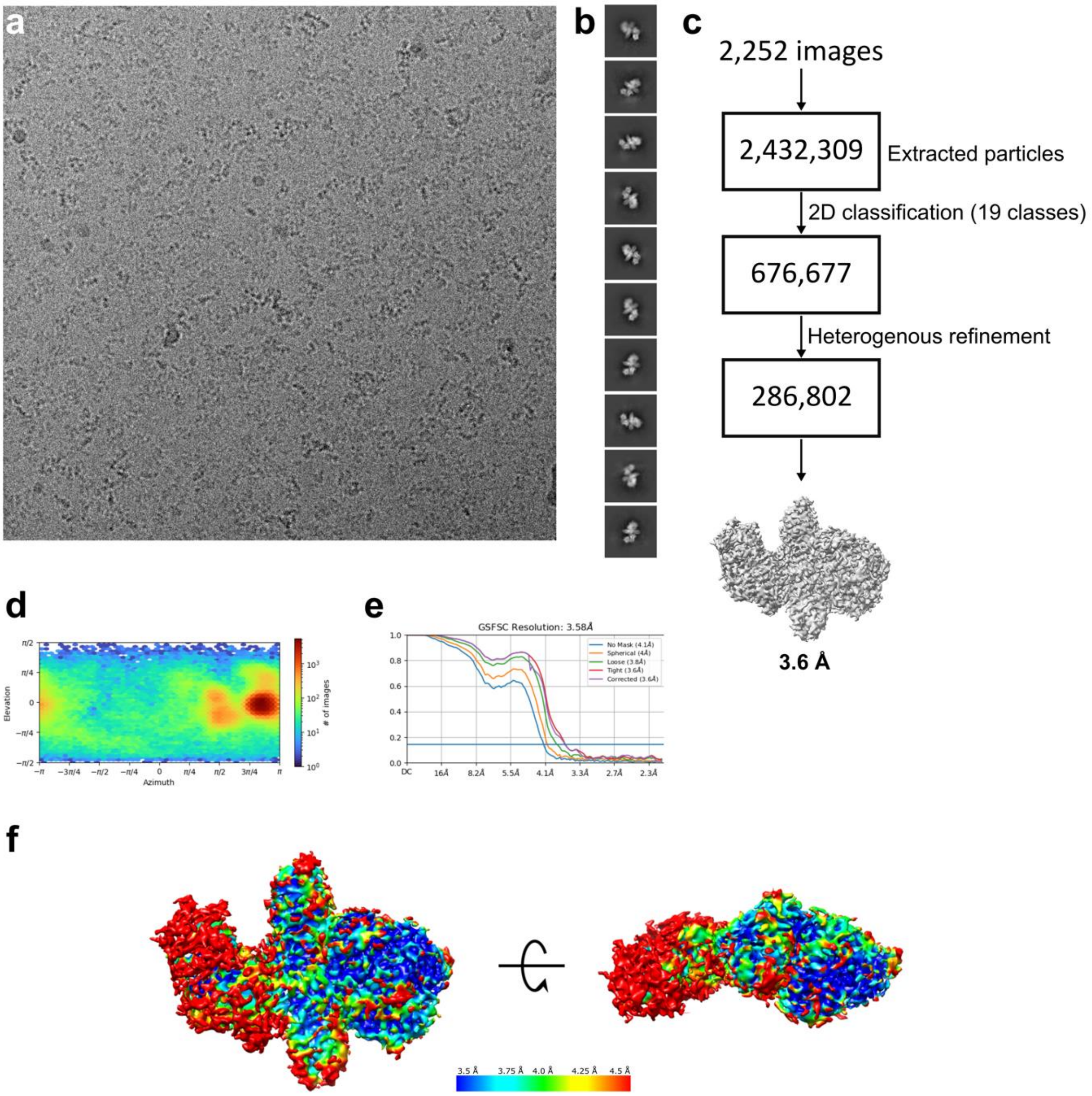

Figure S1. Image processing and evaluation of BoNT/B dataset. **(a)** Representative micrograph from a dataset of 2252 images. **(b)** Representative 2D class averages showing a range of angular orientations. **(c)** Image processing pipeline for BoNT/B, numbers indicate number of particles selected for each reconstruction step. **(d)** Reconstruction and corresponding angular distribution map, indicating BoNT/B has a strong preferred orientation. **(e)** Fourier shell correlation (FSC) curves. **(f)** EM density coloured by local resolution (colour bar indicates local resolution in Å).

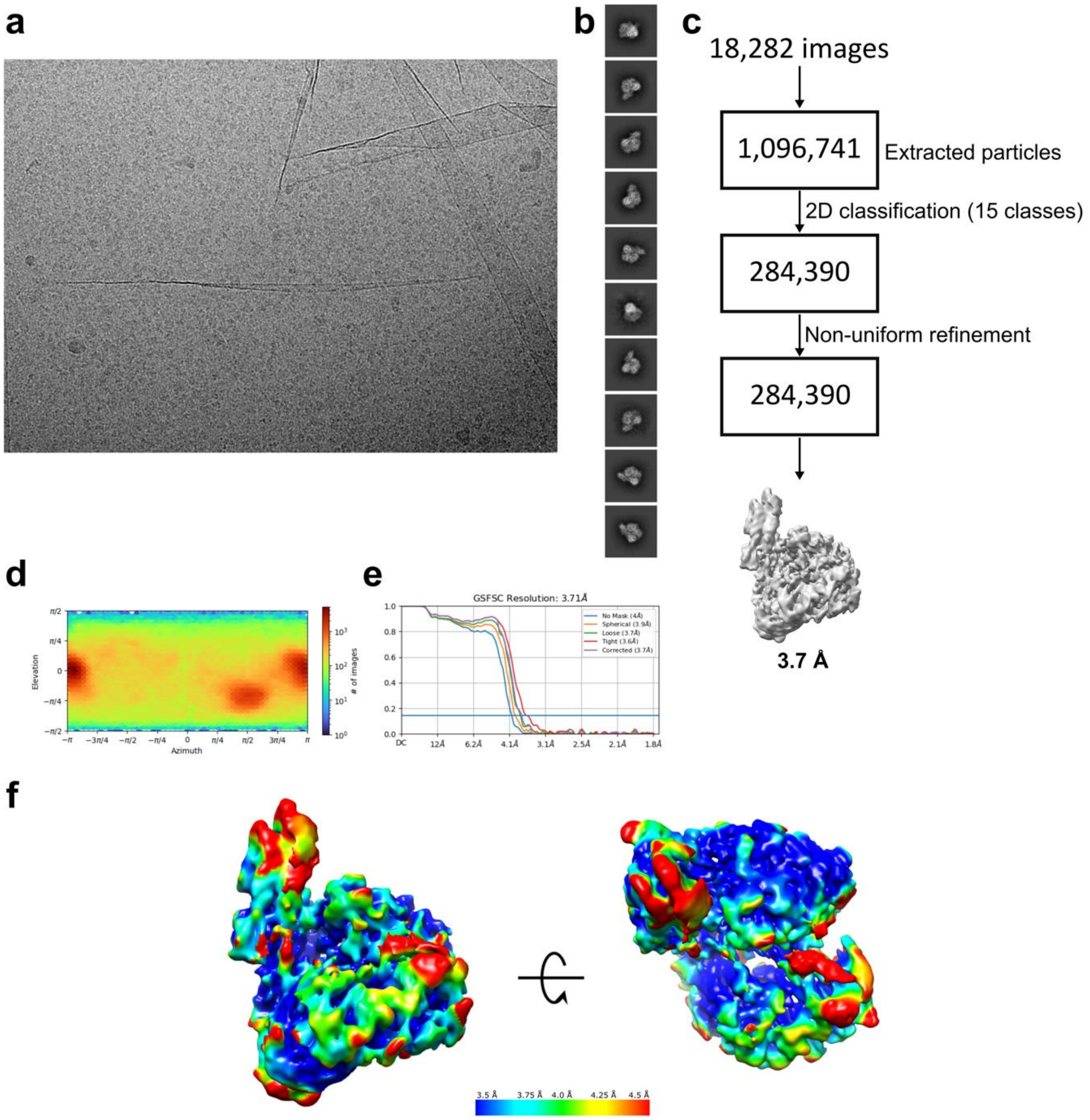

Figure S2. Image processing and evaluation of BoNT/E dataset. (a) Representative micrograph from a dataset of 18282 images. **(b)** Representative 2D class averages showing a range of angular orientations. **(c)** Image processing pipeline for BoNT/E, numbers indicate number of particles selected for each reconstruction step. **(d)** Reconstruction and corresponding angular distribution map, indicating BoNT/B has a strong preferred orientation. **(e)** Fourier shell correlation (FSC) curves. **(f)** EM density coloured by local resolution (colour bar indicates local resolution in Å).

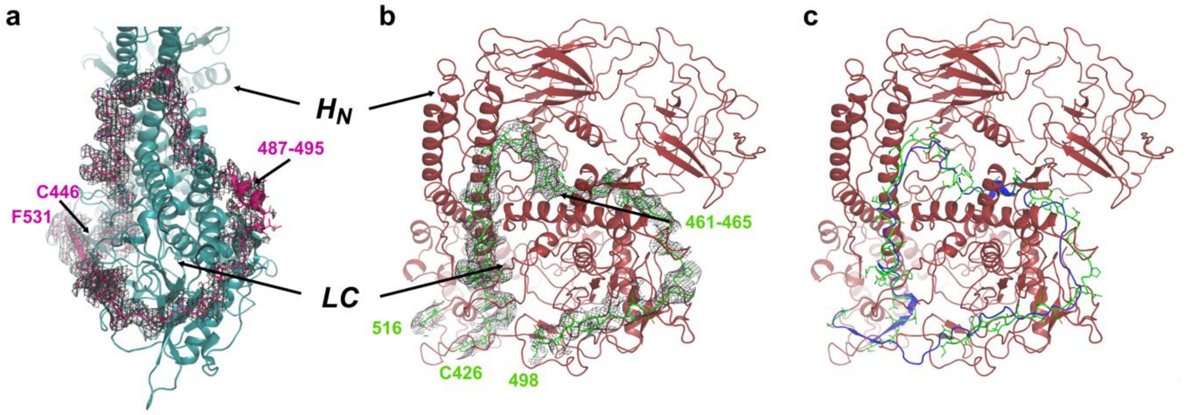

Figure S3. Belt region. (**a**) 3D reconstruction of BoNT/B (teal) and (**b-c**) BoNT/E (red), showing cryo-EM maps around the belt region (in pink and green for /B and /E respectively). Residues 499-515 of BoNT/E could not be modeled. In (**c**) the belt region of the crystal structure is shown in blue (PDB 3FFZ).

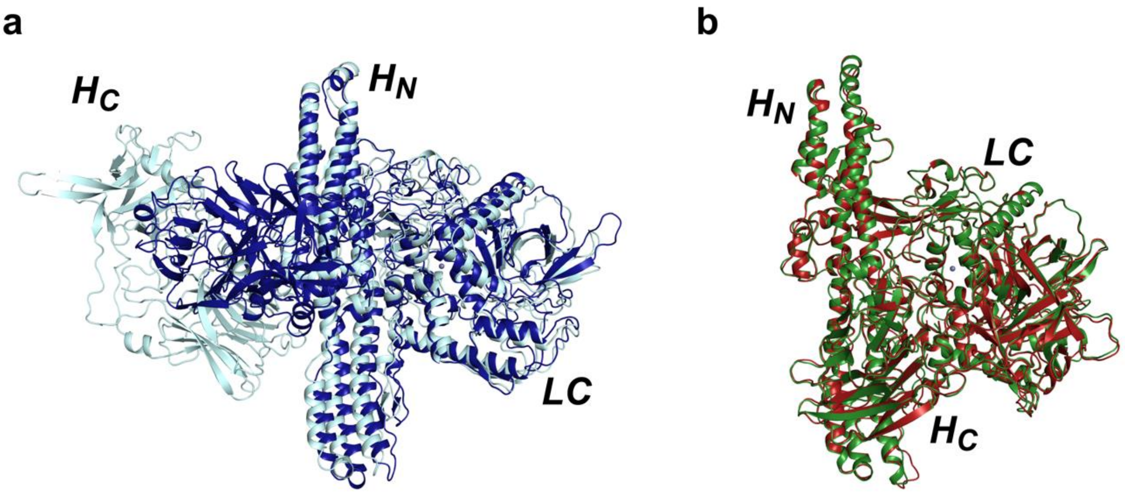

Figure S4. AlphaFold model predictions. (**a**) Superposition of the X-ray crystal structure (cyan) and the AlphaFold-predicted model (blue) of BoNT/B. (**b**) Superposition of the X-ray crystal structure (red) and the AlphaFold-predicted model (green) of BoNT/E. Prediction models were downloaded from the AlphaFold Protein Structure Database [https://alphafold.ebi.ac.uk/entry/B1INP5, and https://alphafold.ebi.ac.uk/entry/Q00496].
